# Efficient Production of Retroviruses Using PLGA/bPEI-DNA Nanoparticles and Application for Reprogramming Somatic Cells

**DOI:** 10.1371/journal.pone.0076875

**Published:** 2013-09-30

**Authors:** Eun Jin Seo, Il Ho Jang, Eun Kyoung Do, Hyo Cheon Cheon, Soon Chul Heo, Yang Woo Kwon, Geun Ok Jeong, Ba Reun Kim, Jae Ho Kim

**Affiliations:** 1 Medical Research Center for Ischemic Tissue Regeneration, Pusan National University School of Medicine, Yangsan, Republic of Korea; 2 Department of Physiology, Pusan National University School of Medicine, Yangsan, Republic of Korea; 3 Research Institute of Convergence Biomedical Science and Technology, Pusan National University School of Medicine, Yangsan, Republic of Korea; University of Kansas Medical Center, United States of America

## Abstract

Reprogramming of somatic cells to pluripotent cells requires the introduction of factors driving fate switches. Viral delivery has been the most efficient method for generation of induced pluripotent stem cells. Transfection, which precedes virus production, is a commonly-used process for delivery of nucleic acids into cells. The aim of this study is to evaluate the efficiency of PLGA/ bPEI nanoparticles in transfection and virus production. Using a modified method of producing PLGA nanoparticles, PLGA/bPEI-DNA nanoparticles were examined for transfection efficiency and virus production yield in comparison with PLGA-DNA, bPEI-DNA nanoparticles or liposome-DNA complexes. After testing various ratios of PLGA, bPEI, and DNA, the ratio of 6:3:1 (PLGA:bPEI:DNA, w/w/w) was determined to be optimal, with acceptable cellular toxicity. PLGA/bPEI-DNA (6:3:1) nanoparticles showed superior transfection efficiency, especially in multiple gene transfection, and viral yield when compared with liposome-DNA complexes. The culture supernatants of HEK293FT cells transfected with PLGA/bPEI-DNA of viral constructs containing reprogramming factors (Oct4, Sox2, Klf4, or c-Myc) successfully and more efficiently generated induced pluripotent stem cell colonies from mouse embryonic fibroblasts. These results strongly suggest that PLGA/bPEI-DNA nanoparticles can provide significant advantages in studying the effect of multiple factor delivery such as in reprogramming or direct conversion of cell fate.

## Introduction

Since the first demonstration of reprogramming of mouse embryonic fibroblasts (MEFs) into induced pluripotent stem (iPS) cells by retroviral delivery of four factors (Oct4, Sox2, Klf4, and c-Myc), a range of alternative approaches for generation of pluripotent cells from somatic cells have been reported [1-3]. Among them, retroviruses have been the most efficient and widely utilized [4]. Approximately 96% of published studies used retrovirus (76%) or lentivirus (20%), which results in integration of viral vectors into the genome, and 4% of non-integrating reprogramming adopted mostly episomal vectors. Therefore, improving methods for production of retrovirus is necessary for efficient reprogramming of somatic cells into iPS.

Nanoparticles - particles in the size range 1-1000 nm – have emerged as a promising tool for gene delivery. Due to their predictable safety profile, high DNA-carrying capacity, increased adaptability, and the simplicity of large-scale production and quality control, nanoparticles have gained popularity [5,6]. They can be sufficiently large to accommodate multiple types of cargo and contain multiple targeting ligands that can direct them to target cells [7]. Poly (D,L-lactide-co-glycolide) (PLGA) is a copolymer of glycolic acid and lactic acid, which are linked by an ester bond. PLGA-based nanoparticles have drawn attention due to their biocompatibility, biodegradability, and sustained-release of cargo [8,9]. Nucleic acid is loaded onto PLGA nanoparticles either by encapsulation or adsorption. Encapsulation provides protection and a controlled-release profile, and adsorption saves the cargo from the harsh process of nanoparticle production. With modifications, the efficiency of encapsulation can increase up to 80%, however, the nucleic acid loading remains low (0.1 to 1 mg per 100 mg nanoparticles) [10,11]. Adsorption offers increased loading and immediate release of nucleic acid, which may improve transfection efficiency [12,13]. Low electrostatic interaction between PLGA and nucleic acid can be circumvented by coating the surface with a cationic excipient such as branched polyethyleneimine (bPEI) [14].

Due to its high cationic-charge-density potential, bPEI is one of the most widely used polycations for delivery of nucleic acids [15,16]. The positively charged amine groups of bPEI are expected to interact with the negatively charged phosphate groups of nucleic acids to produce a neutral or positively charged complex that can enter the negatively charged cell membrane. bPEI is also believed to facilitate nucleic acid delivery by rupturing the endosome before it reaches the lysosome through a proton sponge mechanism [17]. Correlation of the transfection efficiency and cytotoxicity of bPEI with molecular weight has been reported [18,19]. Several strategies have been adopted to avoid cytotoxicity of high molecular weight bPEI, however, they can lower the transfection efficiency or require architectural design of polymers [20,21]. Thus, careful formulation is required for incorporation of bPEI into nanoparticles.

In this study, episomal DNA vectors were adsorbed onto PLGA/bPEI nanoparticles, and the most efficient ratio of PLGA:bPEI:DNA was determined for the optimal transfection in comparison with a conventional liposome-based transfection method. PLGA/bPEI-DNA nanoparticles were applied for production of viruses containing reprogramming factors, and the viral culture supernatant induced successful reprogramming of MEFs into iPS cells. These results strongly suggest that PLGA/bPEI-DNA nanoparticles can provide an excellent platform for introduction of multiple factors into cells.

## Materials and Methods

### Materials

Human adipose tissue-derived mesenchymal stem cells (hASCs) were isolated from subcutaneous adipose tissues, which were obtained from patients undergoing elective surgeries. Written informed consent was obtained from all donors as approved by the Institutional Review Board of Pusan National University Hospital. PLGA Resomer^®^ RG503H (MW ~27 kDa) was supplied from Boehringer Ingelheim (Ridgefield, CT). Methylene chloride (Dichloromethane, analytical grade) was purchased from EMD Millipore (Billerica, MA). Lipofectamine 2000 was obtained from Life Technologies Korea (Seoul, Korea). TE buffer (10 mM Tris/HCL, pH 8.0, 1 mM EDTA) was purchased from Cosmo Genetech (Seoul, South Korea). Cell culture media were purchased from Welgene (Daegu, South Korea). pMX-GFP, pMXs-Oct4, pMXs-Sox2, pMXs-Klf4, pMXs-c-Myc, gag/pol, and VSV.G were obtained from Dr. Jeong Beom Kim (UNIST, South Korea) [22]. p-EGFP, p-EYFP, and p-ECFP were obtained from Clontech Laboratories, Inc. (Mountain View, CA). Branched PEI (bPEI, MW 0.8, 1, 25 kDa), polyvinyl alcohol (MW 30-70 kDa), and all other materials were obtained from Sigma-Aldrich (St Louis, MO).

### Preparation of Nanoparticles

PLGA nanoparticles were prepared using a nanoprecipitation, double emulsification, or modified double emulsification method [23-25]. For encapsulation of DNA, PLGA-DNA nanoparticles were prepared by mixing 100 mg PLGA (in 1 ml of dimethyl sulfoxide for nanoprecipitation and modified double emulsification, or in 1 ml of ethyl acetate for double emulsification) and 100 µg DNA (1 µg/µl in 10 mM Tris/HCL, pH 8.0, 1 mM EDTA). PLGA-DNA nanoparticles were finally prepared at 100 mg/ml in HEPES buffer (20 mM HEPES/NaOH, pH 7.0) for transfection. For generation of PLGA/bPEI-DNA nanoparticles, corresponding volumes of PLGA nanoparticles (100 mg/ml in HEPES buffer) were mixed with bPEI solution (1 mg/ml in HEPES buffer) and incubated for 5 min at room temperature (RT), followed by addition of DNA and incubation for an additional 20 min at RT. For generation of bPEI-DNA complexes, DNA was added to bPEI solution and incubated for 20 min at RT. Details of preparation are summarized in Table S1 and Figure S3. 

### Nanoparticle Characterization

Pictures of nanoparticles were taken by transmission electron microscopy (TEM, H-7600, Hitachi, Nissei, Japan). Nanoparticle size and zeta potential were determined using a Laser Diffraction Particle Size Analyzer (LS 13320, Beckman Coulter, Inc., USA) and an Electrophoretic Light Scattering Spectrophotometer (ELS8000, Otsuka Electronics, Japan).

### Cell culture

Human Embryonic Kidney (HEK) 293FT cells and NIH3T3 cells were maintained in Dulbecco’s modified Eagle’s Medium with 10% fetal bovine serum. Human adipose tissue-derived stem cells were isolated and maintained in α-MEM with 10% fetal bovine serum as described previously [26]. MEFs were prepared as described previously and maintained in DMEM with 10% fetal bovine serum, and murine embryonic stem cells and iPS cells were maintained in DMEM with 10% fetal bovine serum and leukemia inhibitory factor (1000 U/ml) [1]. All other cell lines were cultured as described previously [27-29]. All culture media contained penicillin (0.5 U/ml) streptomycin (50 µg/ml), unless stated otherwise. Cells were maintained at 37°C in a humidified incubator with 5% CO_2._

### Transfection and virus production

Cells were plated with 80% confluence in six-well plates or 100 mm dishes. Cells were switched to a half volume of fresh media containing no antibiotics before transfection. Nanoparticles were incubated with 500 µl of antibiotic free media for 20 min at RT and applied to cells. Media were changed after incubation for 4 h. Transfection with lipofectamine 2000 followed the manufacturer’s protocol. For virus production, HEK293FT cells in 100 mm dishes were transfected with pMX-GFP, pMXs-Oct4, pMXs-Sox2, pMXs-Klf4, or pMXs-c-Myc in combination with gag/pol and VSV.G. The culture supernatants were harvested after 48 h, filtered through a 0.45 µM membrane, and stored at -80°C.

### Reprogramming MEFs into iPS cells

1x10^5^ MEFs were plated in six-well plates and incubated with HEK293FT culture supernatant containing viruses (Oct4, Sox2, Klf4, and c-Myc) for 48 h. Infected MEFs were transferred onto fresh MEFs on day 2 after infection and switched to ES media on day 3. Emergence of colonies was monitored, and individual colonies were picked on day 10 for further culture. Colonies were subjected to alkaline phosphatase staining and immunohistochemistry on day 17.

### Flow cytometry, immunocytochemistry, and fluorescence microscopy

For measurement of GFP expression, cells were analyzed using FACSCanto II (BD Bioscience, San Jose, CA) and FACSDiva software 6.1.3. Fluorescence images were taken by Leica DM IRB (Leica) or FluoView FV1000 (Olympus) and analyzed using ImageJ 1.43u or FV10-ASW ver 3.0. Immunocytochemistry was performed as described previously [30].

## Results

### PLGA/bPEI-DNA complexes are characterized as nanoparticles

**Figure 1:**
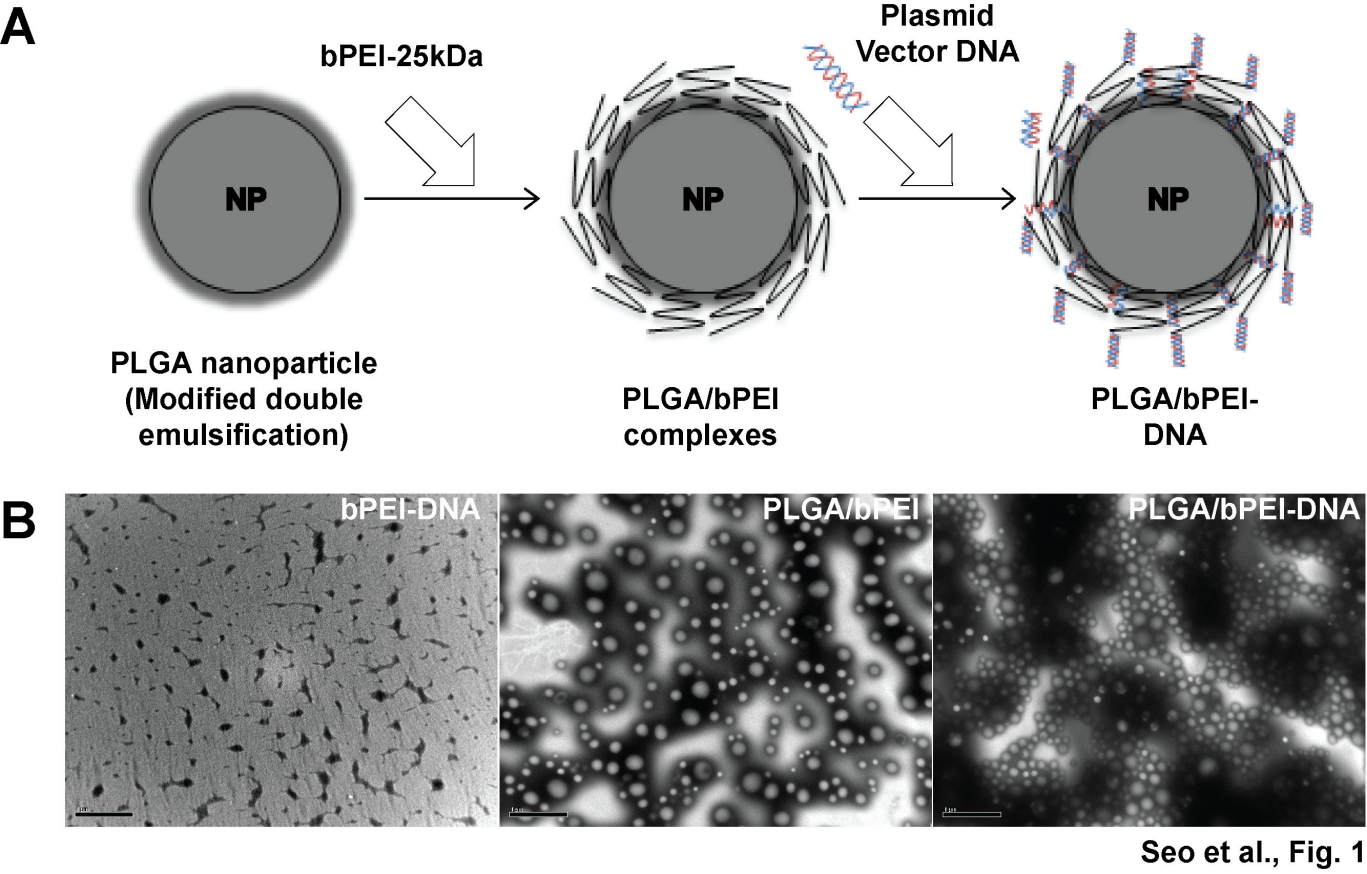
Schematic representation for generation of PLGA/bPEI-DNA nanoparticles. (A) Schematic representation of coating PLGA nanoparticles with cationic 25K bPEI and subsequent loading of DNA is shown. (B) Transmission electron microscopy images of nanoparticles are shown. Scales bars represent 1 µm. n=5.

The efficiency of transfection, which is commonly used for delivery of nucleic acids for numerous purposes, varies in different cell lines, and the optimal condition should be carefully decided. Due to its fast growing and highly transfectable character, the HEK293FT cell line is suitable for testing the efficiency of transfection and virus production. PLGA nanoparticles were generated using a modified double emulsification method followed by bPEI (25 kDa) coating and DNA adsorption (Figure 1). The average diameter of PLGA nanoparticles was 275 nm, and the addition of bPEI and DNA increased the diameter to 282 nm. PLGA nanoparticles exhibited a negative zeta potential (-19.75 mV) due to the carboxyl group (Table 1). bPEI coating increased the zeta potential to 26.97 mV, and PLGA/bPEI-DNA nanoparticles showed a zeta potential of 23.4 mV, which is favorable for the interaction with the negatively charged cell membrane.

**Table 1:** The physicochemical parameters of PLGA nanoparticles prepared by different methods (mean ± SD, *n*=3).

Methods	Average particle size (nm)	Polydispersity index	Zeta potential (mV)
Free PLGA nanoparticles	275	0.051	-19.75±1.47
PLGA NP/bPEI complexes (2:1)	273	0.183	36.97±1.28
PLGA/bPEI-DNA (6:3:1)	282	0.309	23.40±2.39
bPEI-DNA complexes (3:1)	114	0.238	38.55±0.82

### 6:3:1 is the optimal PLGA:bPEI:DNA (w/w/w) ratio for efficient transfection in HEK293FT cells

**Figure 2:**
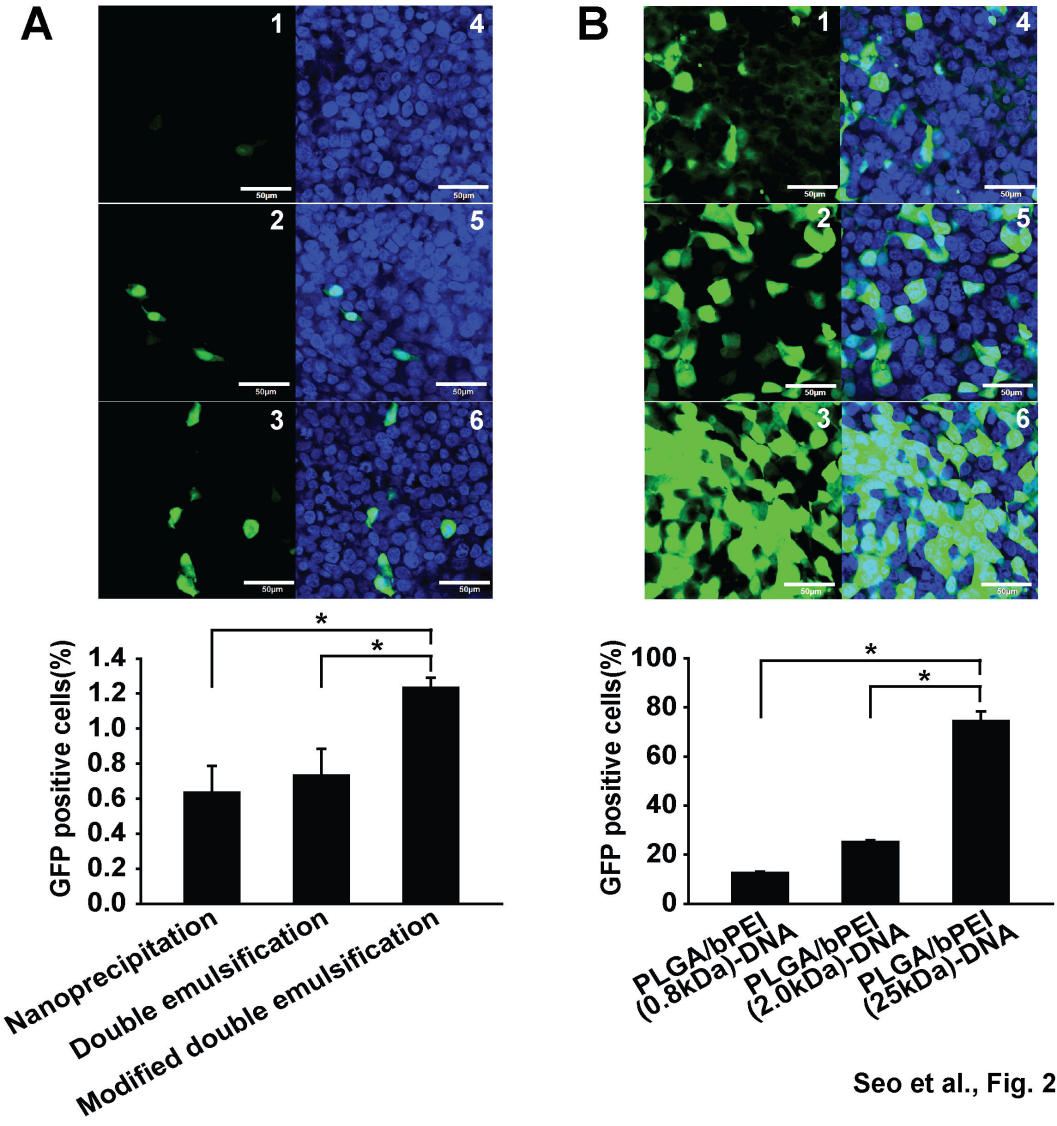
Transfection efficacy of HEK293FT cells using various nanoparticles. GFP positive cells were determined by flow cytometry analysis approximately 24 h (B) or 40 h (A) after transfection. (A) HEK293FT cells were transfected with DNA encoding enhanced green fluorescence protein (EGFP) (pEGFP) using PLGA nanoparticles prepared using different protocols. Bright field images (1-3) and fluorescence images (4-6) of cells transfected with PLGA nanoparticles prepared by nanoprecipitation (1,4), double emulsification (2,5), and modified double emulsification (3,6) protocol are shown. 50 µl of PLGA-DNA preparation (100 mg PLGA and 100 µg DNA / ml) was applied to each well of six-well plates. (B) Bright field images (1-3) and fluorescence images (4-6) of cells transfected with PLGA/bPEI-DNA nanoparticles prepared with different molecular weight bPEIs (1, 4; 0.8 kDa, 2, 5; 2 kDa, 3, 6; 25 kDa), using a modified double emulsification protocol are shown. 1 µg of pEGFP was applied to each well of a six-well plate. Scale bars represent 100 µm (*, p < 0.05 by Student’s t-test). n=3.

**Figure 3:**
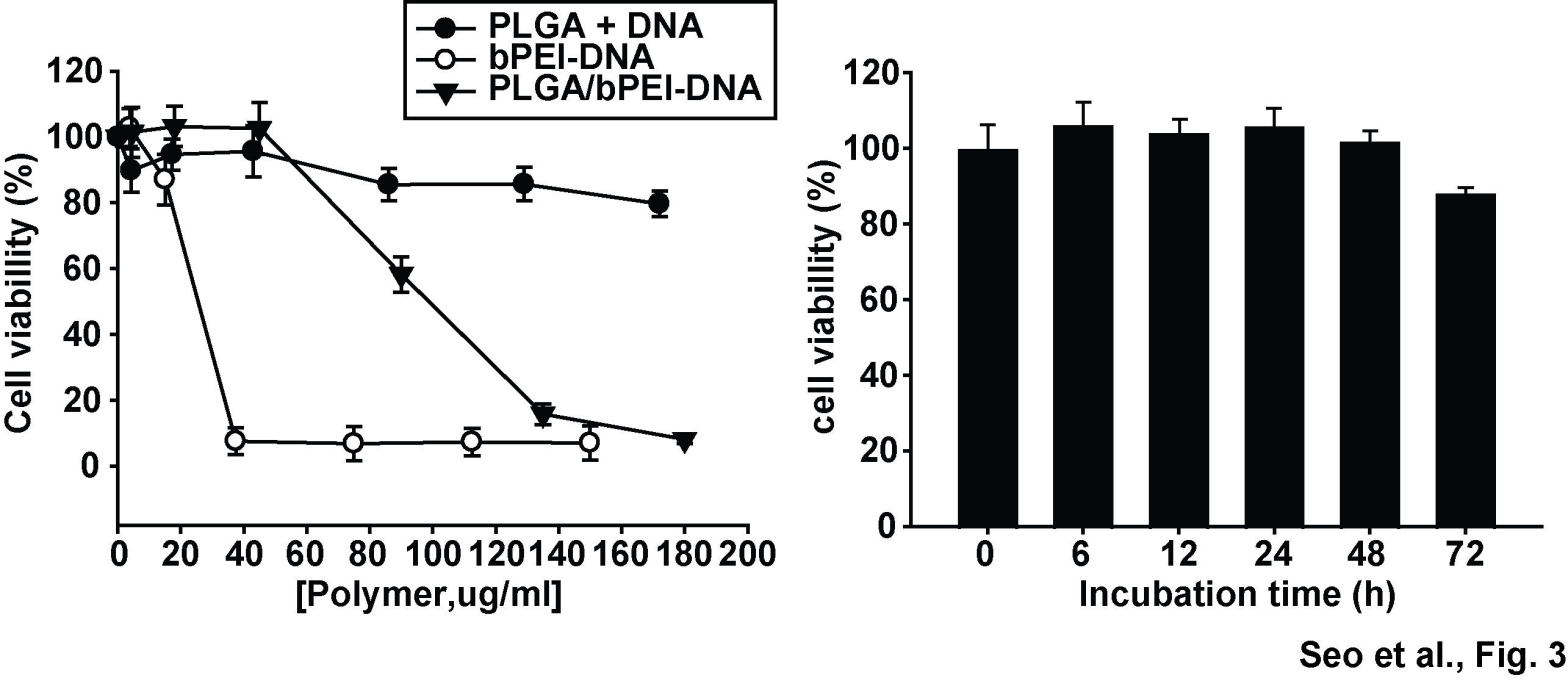
Effects of nanoparticles on viability of HEK293FT cells. Cells in 24-well plate were subjected to MTT assay after incubation with different amounts of PLGA+DNA (DNA adsorbed on PLGA nanoparticles, 6:1, w/w), bPEI-DNA (3:1, w/w), or PLGA/bPEI-DNA (6:3:1, w/w/w) corresponding to the indicated polymer concentrations (PLGA+bPEI) (left panel). On the right panel, cell viability was tested after incubation with PLGA/bPEI-DNA (6:3:1, w/w/w, 24 well tested) for varying amount of time. n=3.

When HEK293FT cells were transfected with the pLVX-EGFP construct, PLGA nanoparticles prepared using a modified double emulsification method showed increased GFP expression, compared with PLGA nanoparticles prepared using a nanoprecipitation or double emulsification method (Figure 2A). The level of GFP expression and the population of GFP-positive cells were very low in cells transfected with PLGA-DNA nanoparticles, however, the addition of basic bPEI resulted in a dramatic increase in both the expression level of GFP and the GFP-positive cell population (Figure 2B). 25 kDa bPEI transfected approximately 80% of the population, whereas low molecular weight bPEIs (0.8, 2 kDa) were less efficient than 25 kDa bPEI (Figure 2B). The cytotoxicity of bPEI-DNA complexes without PLGA was significantly higher than that of PLGA nanoparticles adsorbing DNA. PLGA/bPEI-DNA nanoparticles exhibited significantly improved cell viability, compared with bPEI-DNA complexes (Figure 3).

**Figure 4:**
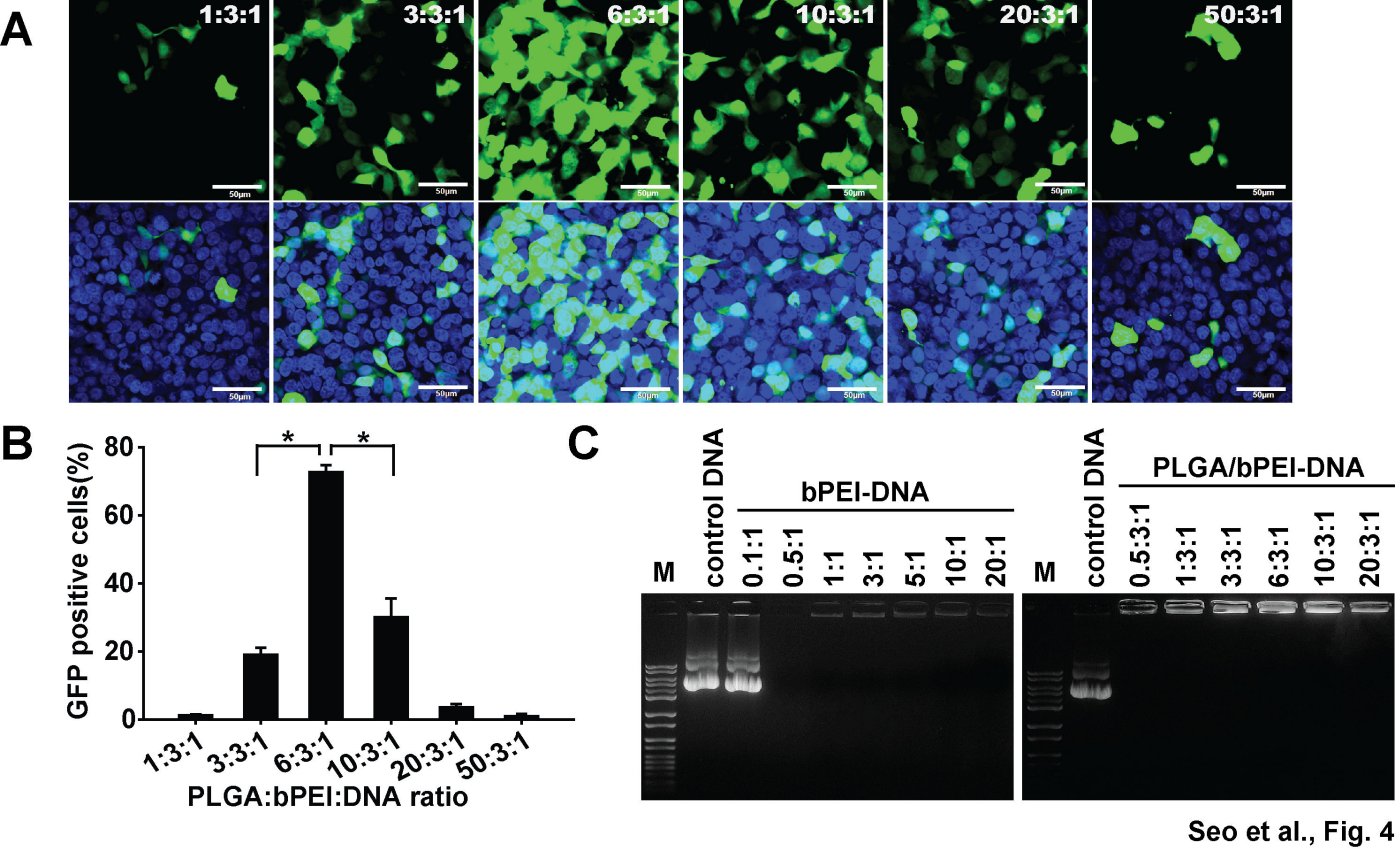
Transfection of HEK293FT cells with pEGFP with varying ratios of PLGA. (A) Fluorescence and bright field images (B) flow cytometry analysis of cells at 48 h after transfection with varying ratios of PLGA/bPEI-DNA (PLGA:bPEI:DNA, w/w/w) nanoparticles are shown. (C) Results of gel electrophoresis analysis of bPEI-DNA (bPEI:DNA, w/w, upper panel) and PLGA/bPEI-DNA (PLGA:bPEI:DNA, w/w/w, lower panel) nanoparticles are shown. 1 µg of DNA was applied to each well (transfection, six-well plate) or lane. Scale bar represents 100 µm (*, p < 0.05 by Student’s t-test). n=3.

**Figure 5:**
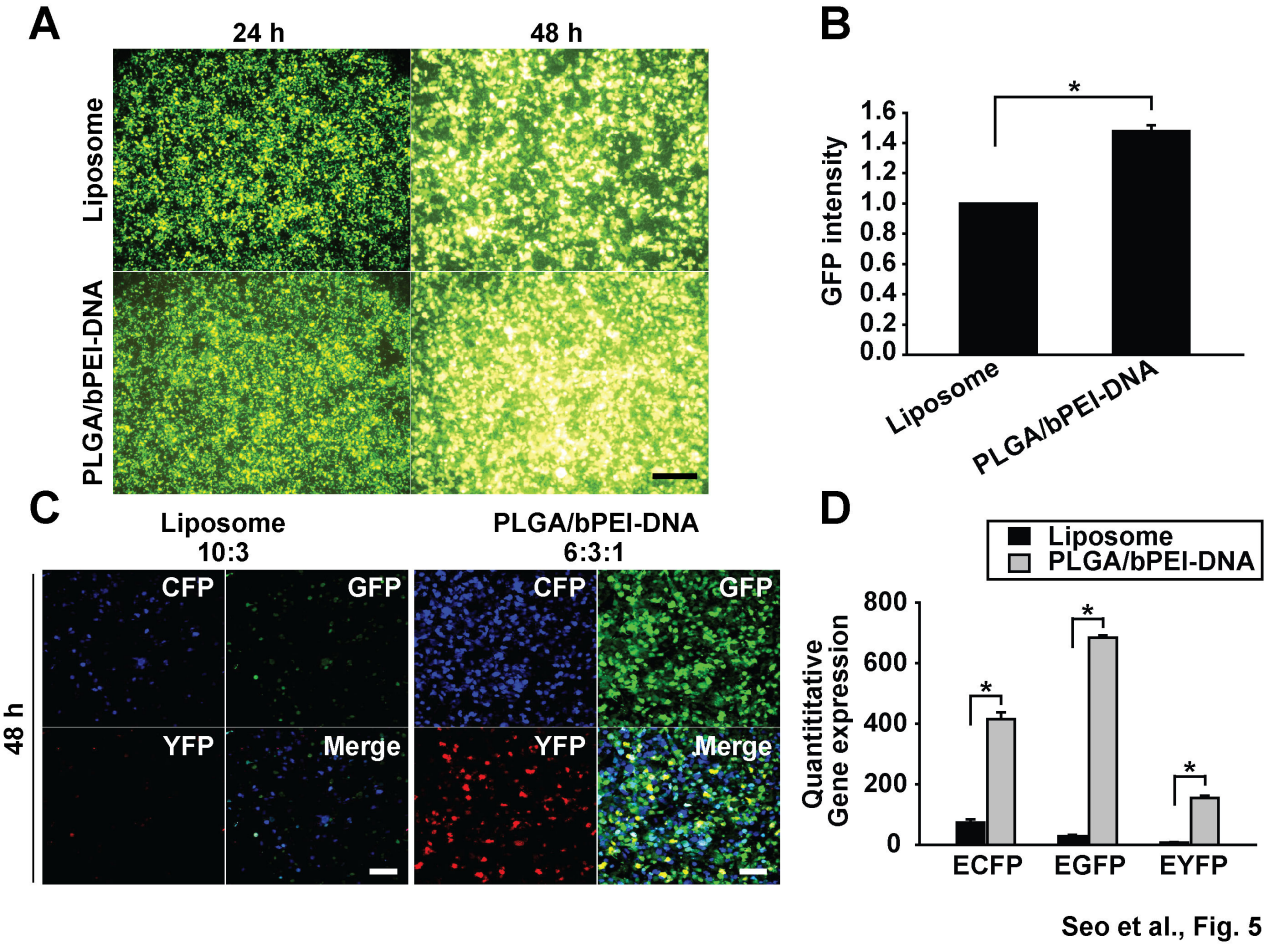
Comparison of transfection efficiency between PLGA/bPEI-DNA (6:3:1, w/w/w) nanoparticles and lipofectamine 2000. (A) Fluorescence images of HEK293FT cells transfected with pEGFP using lipofectamine 2000 or PLGA/bPEI-DNA nanoparticles and (B) quantification of GFP signal are shown (ImageJ ver 1.43u). (C) Confocal microscopy images of HEK293FT cells co-transfected with pECFP, pEGFP, and pEYFP using lipofectamine 2000 or PLGA/bPEI-DNA nanoparticles and (D) quantification of each signal are shown (Olympus FV10-ASW ver 03.01.01.09). 1 µg of each DNA was applied to each well of a 60 mm plate. Scale bars represent 100 µm (*, p < 0.05 by Student’s t-test). n=3.

With bPEI-DNA complexes, different ratios of bPEI to DNA (w/w) were tested, and 3:1 showed higher transfection efficiency than other ratios (Figure S1). After fixing bPEI-DNA ratio to 3:1, varying amounts of PLGA nanoparticles were mixed with bPEI, followed by DNA, and introduced to cells. Among increasing PLGA ratios, a ratio of 6:3:1 (PLGA:bPEI:DNA, w/w/w) showed superior transfection efficiency (Figure 4A and 4B). This result was not due to the difference in the amount of DNA adsorbed to nanoparticles since the gel run showed the exclusion of ethidium bromide due to compaction by bPEI in all ratios, which means that all input DNA molecules bound to nanoparticles under varying conditions (Figure 4C). When compared with a conventional transfection reagent, lipofectamine 2000, PLGA/bPEI-DNA (6:3:1) nanoparticles showed increased expression with a single gene transfection in HEK293FT cells within minimal cytotoxicity range (Figure 5A and 5B, Table S1). The transfection efficiency of PLGA/bPEI-DNA varied in different cell lines; transfection efficiency of PLGA/bPEI-DNA complexes was higher than that of bPEI-DNA and liposome-DNA complexes in HEK293FT cells, MCF7 (breast cancer), A549 (lung cancer), and A2780 and SKOV3 (ovarian cancers), whereas the transfection efficiency was lower than that of liposome-DNA complexes in PC3 and PC3M (prostate cancer) cells (Figure S2). Nanoparticle uptake reached the maximum at 24 h when hMSCs were incubated with PLGA/bPEI-Rhodamine-B-Isothiocyanate (RITC), but the GFP expression by PLGA/bPEI-GFP(DNA) transfection did not show a dramatic change between 6 h and 24 h incubation in 293FT cells (Figure S4). When HEK293FT cells were co-transfected with ECFP, EGFP, and EYFP, nanoparticles showed higher expression of individual genes and a higher population of co-expressing cells (Figure 5C and 5D). These results suggest that PLGA/bPEI-DNA nanoparticles have a significant advantage in transfection of multiple genes.

### PLGA/bPEI-DNA nanoparticles produce higher titer viruses

**Figure 6:**
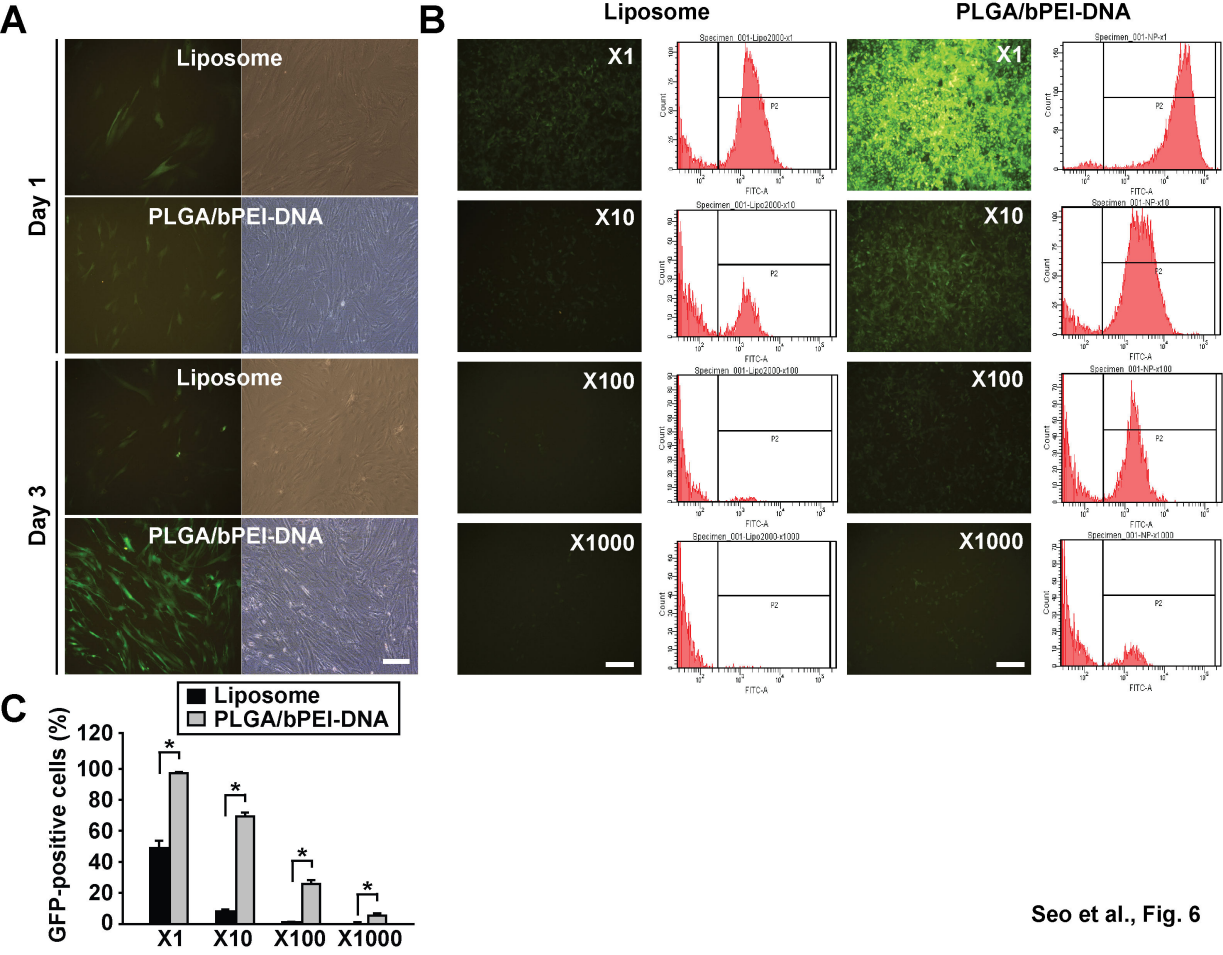
Comparison of the viral yields produced using lipofectamine 2000 or PLGA/bPEI-DNA nanoparticles. HEK293FT cells were co-transfected with pMX-GFP, gag/pol, and VSV-G, and the culture supernatants were harvested at 48 h after transfection. (A) human adipose tissue-derived mesenchymal stem cells were incubated with the viral supernatant (1:1, v/v), and fluorescence and bright field images are shown. n=5. (B, C) NIH 3T3 cells were incubated with the viral supernatant with or without serial dilution (x10, x100, x1000). Fluorescence images and flow cytometry analysis of cells at 48 h after incubation are shown. Scale bars represent 100 µm (*, p < 0.05 by Student’s t-test). Virus infection was tested in 60 mm plates. n=3.

In virus production, co-transfection efficiency is critical for achieving a high viral titer. In comparison with liposome complexes, PLGA/bPEI-DNA nanoparticles were applied for generation of viruses with pMX-GFP. When the same volume of culture supernatants, without concentrating virus particles, was applied to human adipose tissue-derived mesenchymal stem cells, cells transduced with supernatant from nanoparticle transfection showed higher GFP expression (Figure 6A). For comparison of the viral titer, each supernatant was serially diluted and applied to NIH3T3 cells for infection. At a 10-fold dilution, the nanoparticle supernatant infected 8-fold more population than liposome complex supernatant (Figure 6B and 6C). At a 100- and a 1000-fold dilution, liposome complex supernatant did not show a significant infection, however, the nanoparticle supernatant infected 25.7% and 5.3% of the population, respectively. These results demonstrate the superiority of PLGA/bPEI-DNA nanoparticles in achieving a high titer of virus.

### Viruses produced by PLGA/bPEI-DNA nanoparticles reprogram MEFs into iPS cells more efficiently

**Figure 7:**
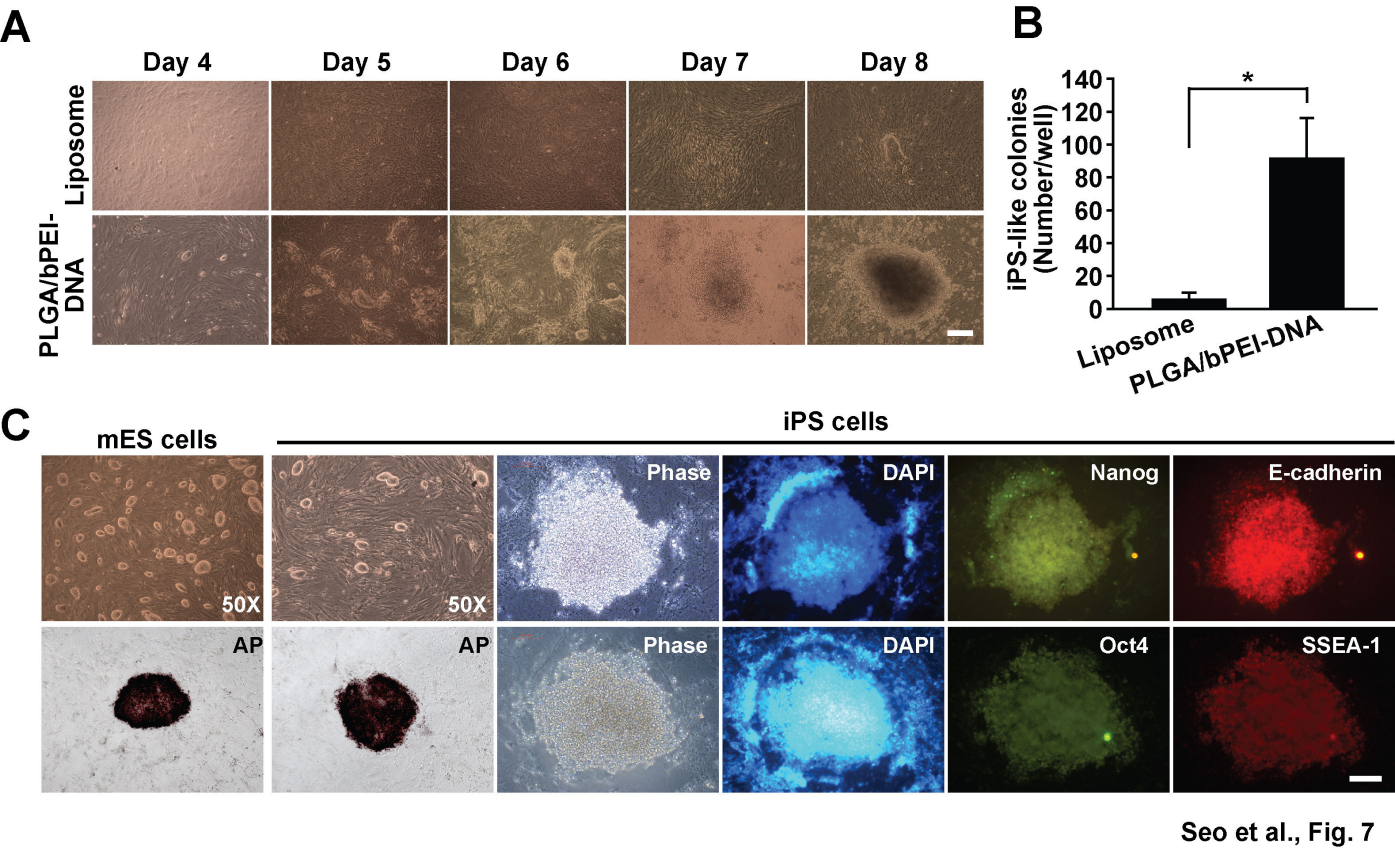
Generation of iPS cells from MEFs using nanoparticle-based production of retrovirus. HEK293FT cells were co-transfected with pMXs-(Oct4, Sox2, Klf4, or c-Myc), gag/pol, and VSV-G using lipofectamine 2000 or PLGA/bPEI-DNA nanoparticles, and the culture supernatants were harvested at 48 h after transfection. (A) MEFs were incubated with the viral supernatant containing reprogramming factors (Oct4, Sox2, Klf4, and c-Myc) (1:1, v/v). Bright field images of time course monitoring are shown. (B) Colonies were counted on day 10 after virus infection. (C) Colonies at day 17 after infection were tested for expression of alkaline phosphatase, Nanog, E-cadherin, Oct4, or SSEA-1. Bright field and fluorescence images of colonies are shown. Scale bars represent 100 µm (*, p < 0.05 by Student’s t-test). n=3.

Nuclear reprogramming is currently an active area of research, and viruses have been adopted for delivery of reprogramming genes, such as Oct4, Sox2, Klf4, and c-Myc [31-34]. PLGA/bPEI-DNA nanoparticles were applied for generation of viruses containing these reprogramming genes in comparison with liposome-DNA complexes. When each culture supernatant was mixed and applied to MEFs, nanoparticle supernatant mixture generated microcolonies on day 4 after infection, whereas liposome-DNA complex supernatant mixture produced detectable colonies on day 8 (Figure 7A). When colonies were counted on day 10 after infection, viral supernatant from PLGA/bPEI-DNA nanoparticles generated far more colonies (Figure 7B). Colonies were cultured until day 17 and subjected to staining of embryonic stem cell marker expression. As shown in Figure 7C, colonies were positive for alkaline phosphatase, E-cadherin, SSEA-1, Oct4, and Nanog. These results strongly suggest that PLGA/bPEI-DNA nanoparticles produced a high titer of functional viruses, which resulted in successful reprogramming of MEFs into iPS cells.

## Discussion

Due to its remarkable DNA condensation ability, lysosomal buffering capacity, protection of DNA from degradation, and effectiveness both in tissue culture and *in vivo*, bPEI has drawn attention in gene therapy as a non-viral carrier [15,16]. High molecular weight bPEIs with a molecular weight greater than 25 kDa have shown higher transfection efficiency but also higher cytotoxicity [18,19]. PLGA/bPEI-DNA nanoparticles with lower molecular weight bPEIs did not show as strong GFP expression as nanoparticles with 25 kDa bPEI; however, cytotoxicity of 25 kDa bPEI alone with DNA was quite significant. Incorporation of PLGA nanoparticles into bPEI-DNA complexes resulted in significantly increased cell viability, probably due to delayed release of toxic free bPEI, which can result in formation of aggregation on cell membranes and cause mitochondrial damage [35]. Increased proton sponge effect could also have contributed by expediting the escape of nanoparticles from the endosomes before they reach lysosomes, thus decreasing the damage from lysosomal breakdown [17].

In the present study, we showed that the optimal ratio of PLGA:bPEI:DNA was 6:3:1 (w/w/w) in HEK293FT cells. This can be converted to an N/P ratio (polymer nitrogen to DNA phosphate) of 23.26 [36]. Findings from a recent study showed that, in HePG2 cells, an N/P ratio of 6 was optimal for expression of miRNA with PLGA/bPEI-DNA nanoparticles [37]. The optimal N/P ratio may vary from cell line to cell line. With bPEI-DNA complexes, the optimum N/P ratio was 3.3 in COS-1 cells, 16.7 in Calu-3 cells, and between 10 and 20 in aerosol delivery into mouse lung [36,38]. In comparison with liposome-DNA complexes, PLGA/bPEI-DNA (6:3:1) nanoparticles showed superior transfection efficiency in breast, lung, and ovarian cancer cell lines, but not in prostate cancer cell lines (Figure S2). Readjustment of PLGA:bPEI:DNA ratio in different cell types may be needed in order to obtain the optimal transfection efficiency.

In the present study, we showed that the efficiency of multiple gene transfection using PLGA/bPEI-DNA nanoparticles was higher than that of liposome-DNA complexes. The higher capacity of nanoparticles for carrying DNA molecules may be responsible for the higher transfection efficiency. Non-linear transfection efficiency was reported when the liposome complexes were prepared at a low concentration [39]. Without increasing liposomes, tripling the DNA for co-transfection could have contributed to the decrease of transfection efficiency. Small liposome complexes can deliver a small amount of DNA to many cells; however, large nanoparticles can deliver a larger amount of DNA to fewer cells. The difference in the net result can be significant when multiple genes are co-transfected. For virus production, where more than three different constructs are usually transfected, nanoparticles showed a significant advantage over liposomes.

This advantage was demonstrated in generation of iPS cells using viruses produced by nanoparticles. Incubation of MEFs with the culture supernatant of HEK293FT cells transfected with viral constructs resulted in detection of small, round colonies on day 4 after incubation with PLGA/bPEI-DNA supernatant, whereas the first colonies were found on day 8 with culture supernatant of HEK293FT cells transfected with viral constructs using liposome-DNA complexes (Figure 7). In the previous time course studies of mouse iPS cell generation, the first small colonies were detected on day 3 after infection of cells with concentrated viruses and on day 9 with the culture supernatant [33,34]. Although other factors could have contributed to the difference, our results demonstrate that PLGA/bPEI-DNA nanoparticles produce functional viruses in high titer. Since the first report on generation of iPS cells, various delivery approaches have been reported, however, to date, most publications on reprogramming human somatic cells still use retroviruses (~76%), followed by lentiviruses (~20%) and non-integrating episomal vectors (~4%) [3]. PLGA/bPEI-DNA nanoparticles did not show efficient transfection with MEFs, however, they were superior in cell lines originating from breast cancer or colon cancer, which suggests that PLGA/bPEI-DNA can have an advantage in reprograming certain types of cells using episomal vectors. Episome-mediated and mRNA-mediated reprogramming can produce iPS cells without the integration of foreign genetic materials [40-43]. Testing the efficiency of nanoparticle in episom-mediated or mRNA-mediated transfection can be an interesting future topic. In reprogramming or direct conversion of cell fate, the detailed molecular mechanisms and the role of new factors are under active investigation [44]. PLGA/bPEI-DNA nanoparticles can provide significant advantages in production of virus vectors or delivery of episomal vectors or mRNAs for testing of various factors.

## Conclusions

6:3:1 ratio of PLGA/bPEI-DNA was the most efficient in transfection of HEK293FT cells, and nanoparticles of this ratio showed superior transfection efficiency in many different, but not all, cell lines. PLGA/bPEI-DNA nanoparticles were far superior in multiple gene transfection, which can be beneficial to production of viruses. These results demonstrate that PLGA/bPEI-DNA nanoparticles can be an excellent method for multiple gene delivery.

## Supporting Information

Figure S1 **Transfection of HEK293FT cells with pEGFP using varying ratios of bPEI-DNA (w/w) nanoparticles.** Fluorescence and bright field images of cells at 24 hr after transfection are shown.
(TIF)

Figure S2 **Transfection efficiencies of different nanoparticles in various cell lines.** Cells were transfected with pEGFP using lipofectamine 2000, bPEI-DNA, or PLGA/bPEI-DNA nanoparticles. GFP positivity was analyzed by flow cytometry at 48 hr after transfection.
(TIF)

Figure S3 **Step-wise protocol for preparation of nanoparticles and transfection.**
(TIF)

Figure S4 **Laser scanning confocal microscopy (LSCM) of hMSC transfected with Rhodamine-B-Isothiocyanate (RITC) complexed with PLGA/bPEI nanoparticles.** Cellular uptake profile of PLGA/bPEI-RITC nanoparticles as a function of incubation time (PLGA:bPEI=6:3; 36 µg: 18 µg, 37°C, 6 well tested) is shown. hMSC were incubated with PLGA/bPEI-RITC nanoparticles for indicated time and subjected to LSCM. The inserts in 6 h and 24 h show GFP expression when 293FT cells were transfected with PLGA/bPEI-DNA(GFP) and the indicated incubation time for transfection. GFP images were taken at 24 h after the transfection procedure.
(TIF)

Table S1 **Amount/Concentration of PLGA/bPEI-DNA polymer.**
(DOC)
