# Supplementary figures and images for: Efficient Production of Retroviruses Using PLGA/bPEI-DNA Nanoparticles and Application for Reprogramming Somatic Cells
Source: PLoS One. 2013 Sep 30;8(9):e76875. doi: 10.1371/journal.pone.0076875 (PMC3786964; doi:10.1371/journal.pone.0076875)

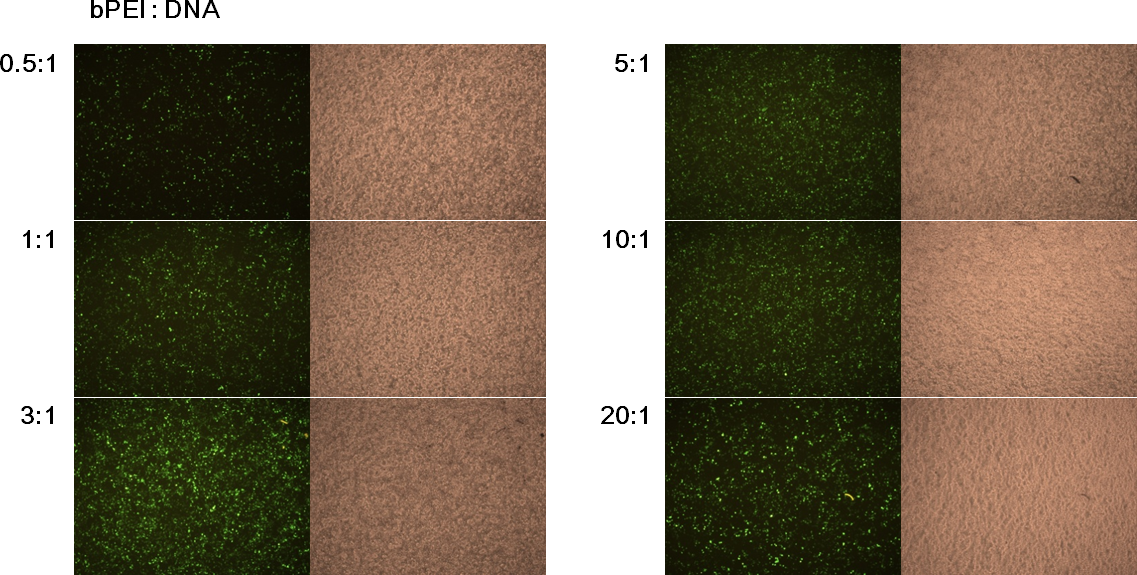

Supplement: Figure S1 — Transfection of HEK293FT cells with pEGFP using varying ratios of bPEI-DNA (w/w) nanoparticles. Fluorescence and bright field images of cells at 24 hr after transfection are shown. (TIF) [file pone.0076875.s001.tif]

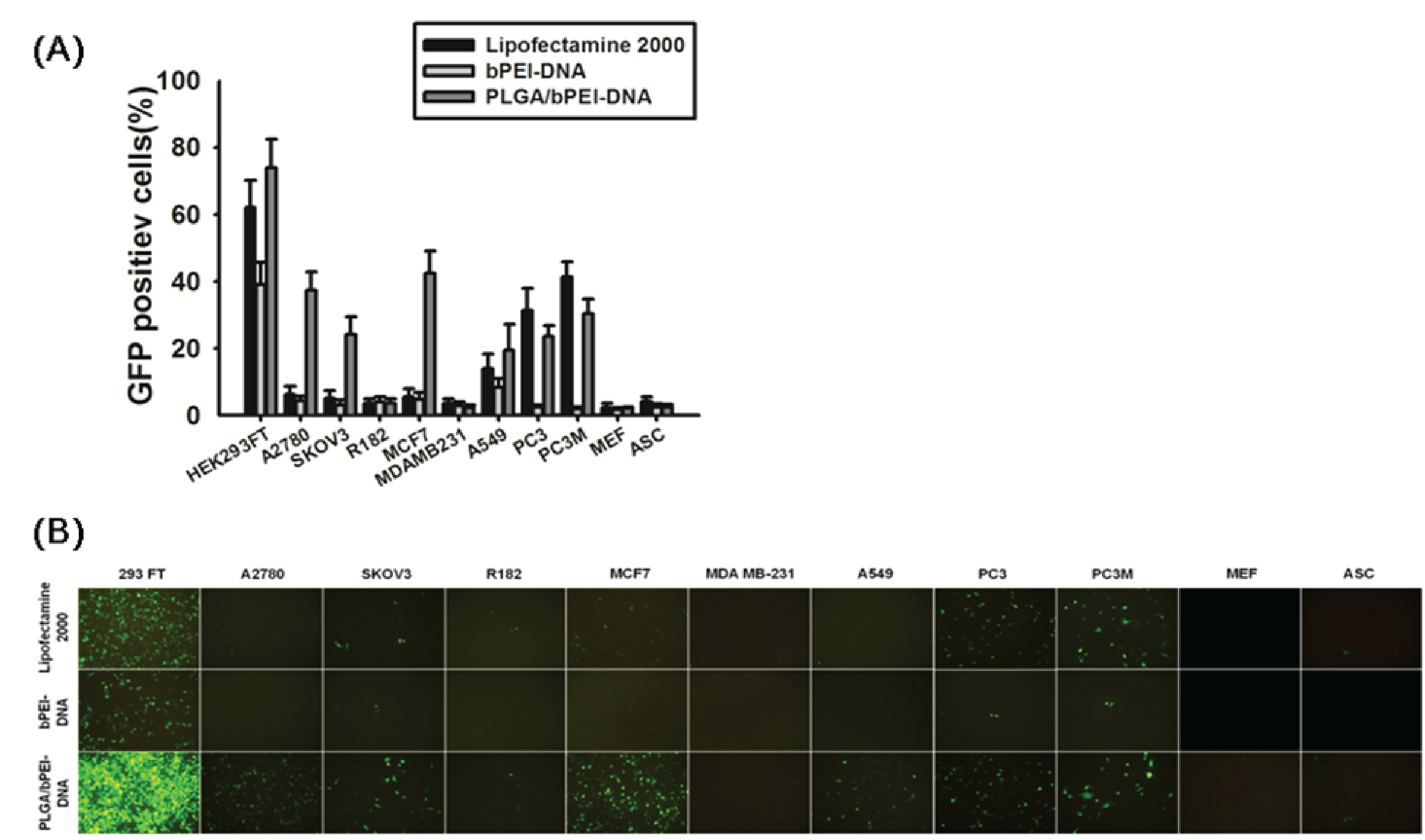

Supplement: Figure S2 — Transfection efficiencies of different nanoparticles in various cell lines. Cells were transfected with pEGFP using lipofectamine 2000, bPEI-DNA, or PLGA/bPEI-DNA nanoparticles. GFP positivity was analyzed by flow cytometry at 48 hr after transfection. (TIF) [file pone.0076875.s002.tif]

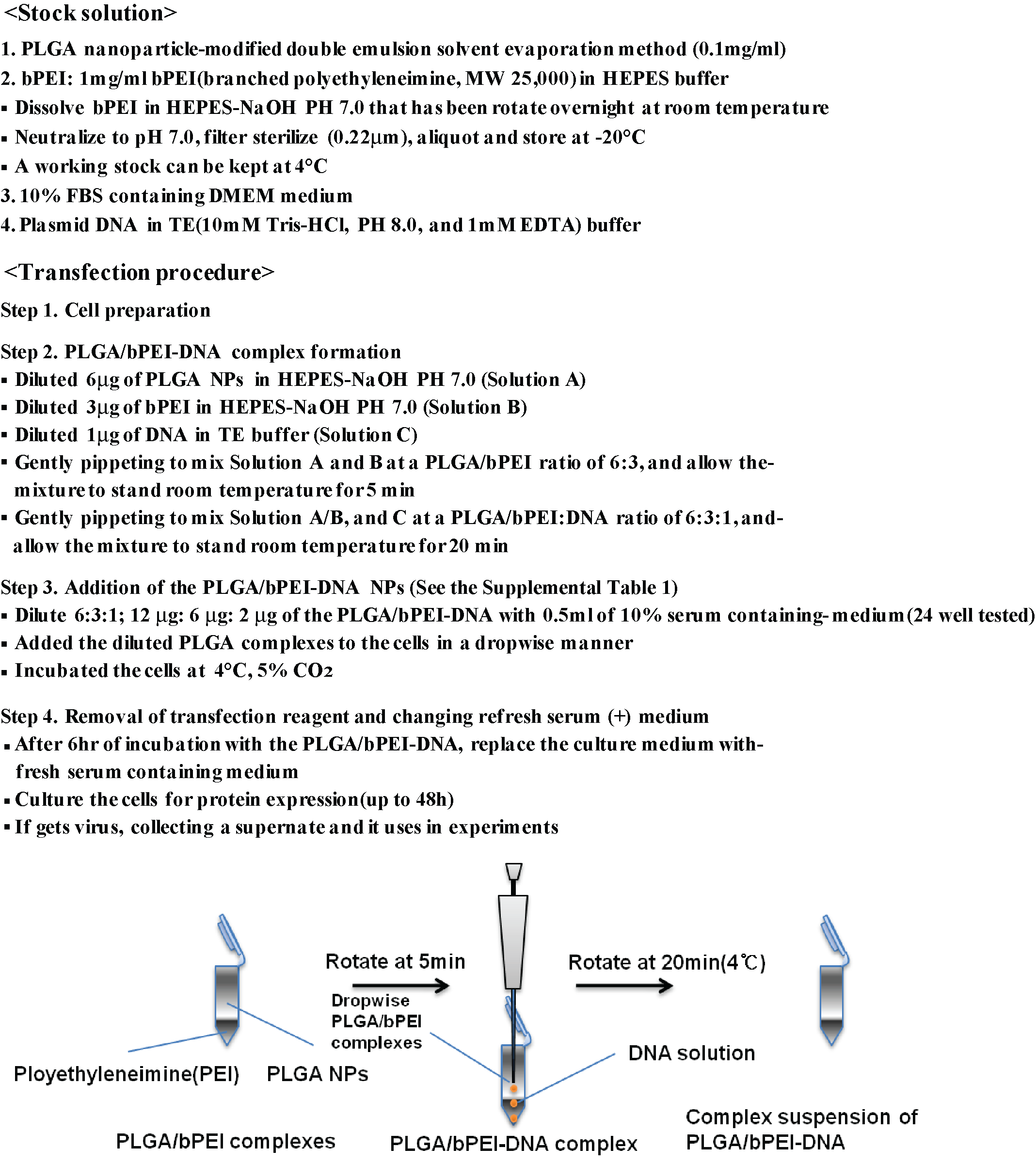

Supplement: Figure S3 — Step-wise protocol for preparation of nanoparticles and transfection. (TIF) [file pone.0076875.s003.tif]

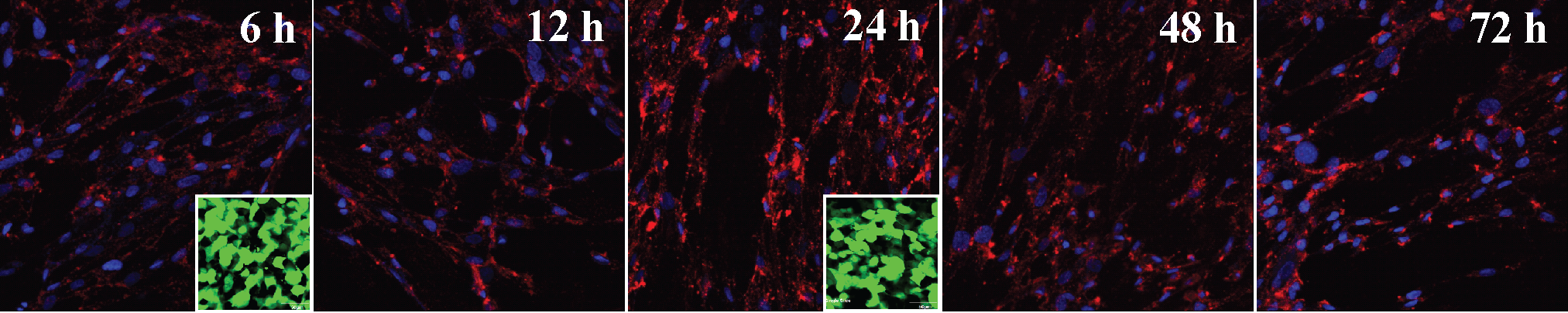

Supplement: Figure S4 — Laser scanning confocal microscopy (LSCM) of hMSC transfected with Rhodamine-B-Isothiocyanate (RITC) complexed with PLGA/bPEI nanoparticles. Cellular uptake profile of PLGA/bPEI-RITC nanoparticles as a function of incubation time (PLGA:bPEI=6:3; 36 µg: 18 µg, 37°C, 6 well tested) is shown. hMSC were incubated with PLGA/bPEI-RITC nanoparticles for indicated time and subjected to LSCM. The inserts in 6 h and 24 h show GFP expression when 293FT cells were transfected with PLGA/bPEI-DNA(GFP) and the indicated incubation time for transfection. GFP images were taken at 24 h after the transfection procedure. (TIF) [file pone.0076875.s004.tif]
