# Supplementary material for: Efficient Production of Retroviruses Using PLGA/bPEI-DNA Nanoparticles and Application for Reprogramming Somatic Cells
Source: PLoS One. 2013 Sep 30;8(9):e76875. doi: 10.1371/journal.pone.0076875 (PMC3786964; doi:10.1371/journal.pone.0076875)
Supplement: Table S1 — Amount/Concentration of PLGA/bPEI-DNA polymer. (DOC) [file pone.0076875.s005.doc]

**Table S1. Amount/Concentration of PLGA/bPEI-DNA polymer**

| **Culture vessel** | **Amount of PLGA/bPEI**  **(6:3, μg/μg)** | **Amount of DNA (μg)** | **Vol. of**  **total medium** | **Concentration of PLGA/bPEI-DNA (PLGA+bPEI+DNA μg/ml)** |
| --- | --- | --- | --- | --- |
| **96 well** | 3/1.5 | 0.5 | 100 μl | 50 |
| **24 well** | 12/6 | 2 | 500 μl | 40 |
| **12 well** | 18/9 | 3 | 1 ml | 30 |
| **6 well** | 36/18 | 6 | 2 ml | 30 |
| **60 mm** | 54/27 | 9 | 4 ml | 22.5 |
| **10 cm** | 72/36 | 12 | 10 ml | 12 |

**Fig. 2A**; Encapsulation; PLGA:DNA,100mg:100μg/ml, 60 μl was added to 2ml of media, **6 well tested.**

**For generation of PLGA/bPEI-DNA (6:3:1, w/w/w) nanoparticles;**

The sonication product of PLGA, 100 mg/ml, was diluted with HEPES buffer to be 10 mg/ml and stored as the stock. For each experiment, 10 mg/ml PLGA stock was 100-fold diluted to be 0.1 mg/ml, and the corresponding volume was mixed with bPEI and DNA. For example, for 6 well test, 360 μl of PLGA (0.1 mg/ml), 18 μl of bPEI (1 μg/μl) and 6 μl of DNA (1 μg/μl) were mixed and added to 2 ml of media.

**Fig. 2B**; Adsorption; PLGA:bPEI:DNA (6:3:1; 36 μg:18 μg:6 μg); **6 well tested.**

**Fig. 3A**; Cell viability (MTT assay); different amount of PLGA+DNA(6:1,w/w), bPEI-DNA(3:1, w/w), PLGA/bPEI-DNA(6:3:1, w/w/w) corresponding to various concentrations of polymer (PLGA + bPEI, μg/ml) were added to 500 μl of media; **24 well tested.**

**Fig. 3B**; Cell viability (MTT assay); PLGA/bPEI-DNA (6:3:1; 12 μg:6 μg: 2 μg); **24 well tested.**

**Fig. 5A**; lipofectamine 2000:DNA (20 μl:8 μg), PLGA/bPEI-DNA (6:3:1; 54 μg:27 μg: 9 μg); **6 mm tested.**

**Fig. 5C**; lipofectamine 2000:DNA (10 μl:3 μg), PLGA/bPEI-DNA (6:3:1; 18 μg:9 μg: 3 μg); **12 well tested.**

**Fig. 6C**; lipofectamine 2000:DNA (pMX-GFP, gag/pol, VSV-G), (30 μl:(6 μg+3 μg+3 μg)), PLGA/bPEI-DNA (6:3:1; 72 μg:36 μg:(6 μg+3 μg+3 μg)), **10 cm tested for virus production.**
